# Supplementary material for: The Intrinsic Fragility of the Liquid–Vapor Interface: A Stress Network Perspective
Source: Langmuir. 2022 Apr 6;38(15):4669–79. doi: 10.1021/acs.langmuir.2c00201 (PMC9022435; doi:10.1021/acs.langmuir.2c00201)
Supplement: Supplementary file 1 — la2c00201_si_001.pdf [file la2c00201_si_001.pdf]

Supporting Information: The Intrinsic Fragility of the Liquid-Vapor Interface: A Stress Network Perspective

Muhammad Rizwanur Rahman,*,† Li Shen,† James P. Ewen,† Daniele Dini,† and E. R. Smith‡

†*Department of Mechanical Engineering, Imperial College London, South Kensington Campus, London SW7 2AZ, United Kingdom*

‡*Department of Mechanical and Aerospace Engineering, Brunel University London, Uxbridge UB8 3PH, United Kingdom*

E-mail: m.rahman20@imperial.ac.uk

Fractal dimension:

The instantaneous configurational stresses are shown in Fig. S1 (a). For the fractal analysis, the positive stresses are filtered out in panel (b). Later, using appropriate thresholds (discussed in the section 'Surface fractals' of the manuscript), the color map in panel (b) is converted into a binary network as in panel (c). The Minkowski dimension is then found by plotting the number of grids, $N(\epsilon)$ required to cover the network against the grid size, ϵ, as in panel (d) and obtaining the slope of the best fit line. The mean of such dimensions of all the instantaneous stress networks are then averaged and reported along with the standard error of the mean (with a magnification factor of 2 for visualization purpose).

The stress maps are more space filling for the low temperature surface, as in the top panels

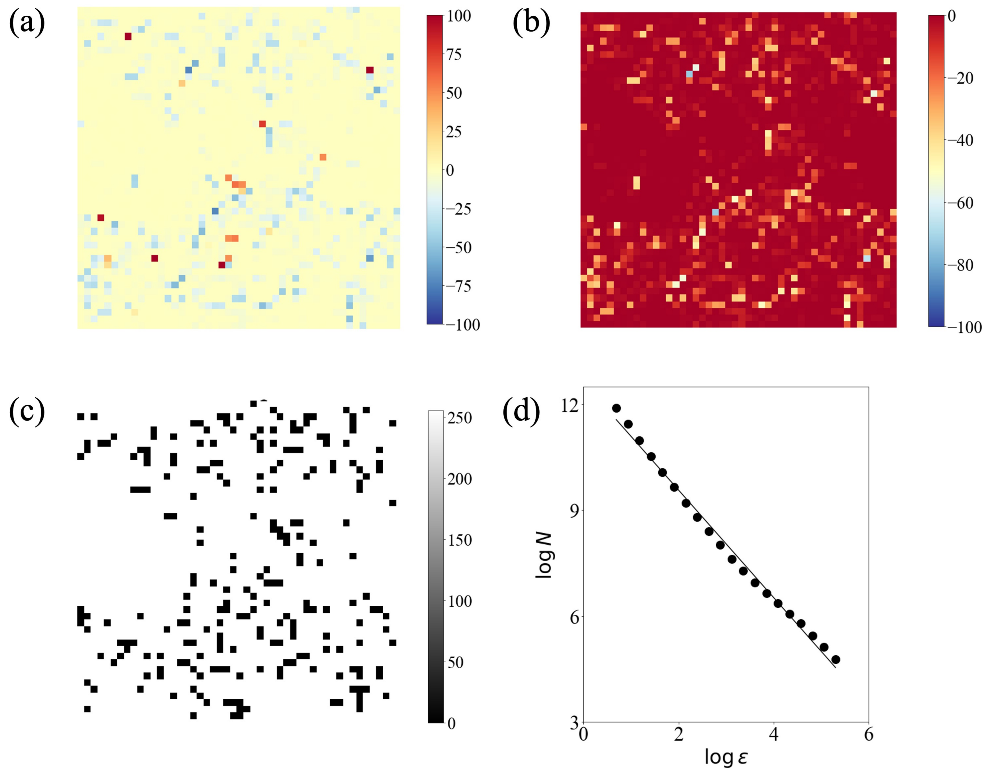

Figure S1: Fractal analysis steps: (a) Instantaneous configurational stress distribution, (b) only the negative configurational stresses of panel a are mapped, (c) color map of panel b is converted into a binary image, (d) Number of grids plotted against the grid size. Negative of the slope of the fitting line (black line) gives the fractal dimension.

of Fig. S2 (a-e), as compared to those for a higher temperature surface as in the bottom panels (f-j). The fractal dimension, Φ, is measured for all such instantaneous network maps and then averaged. Movie SI-1 shows more instances of these networks.

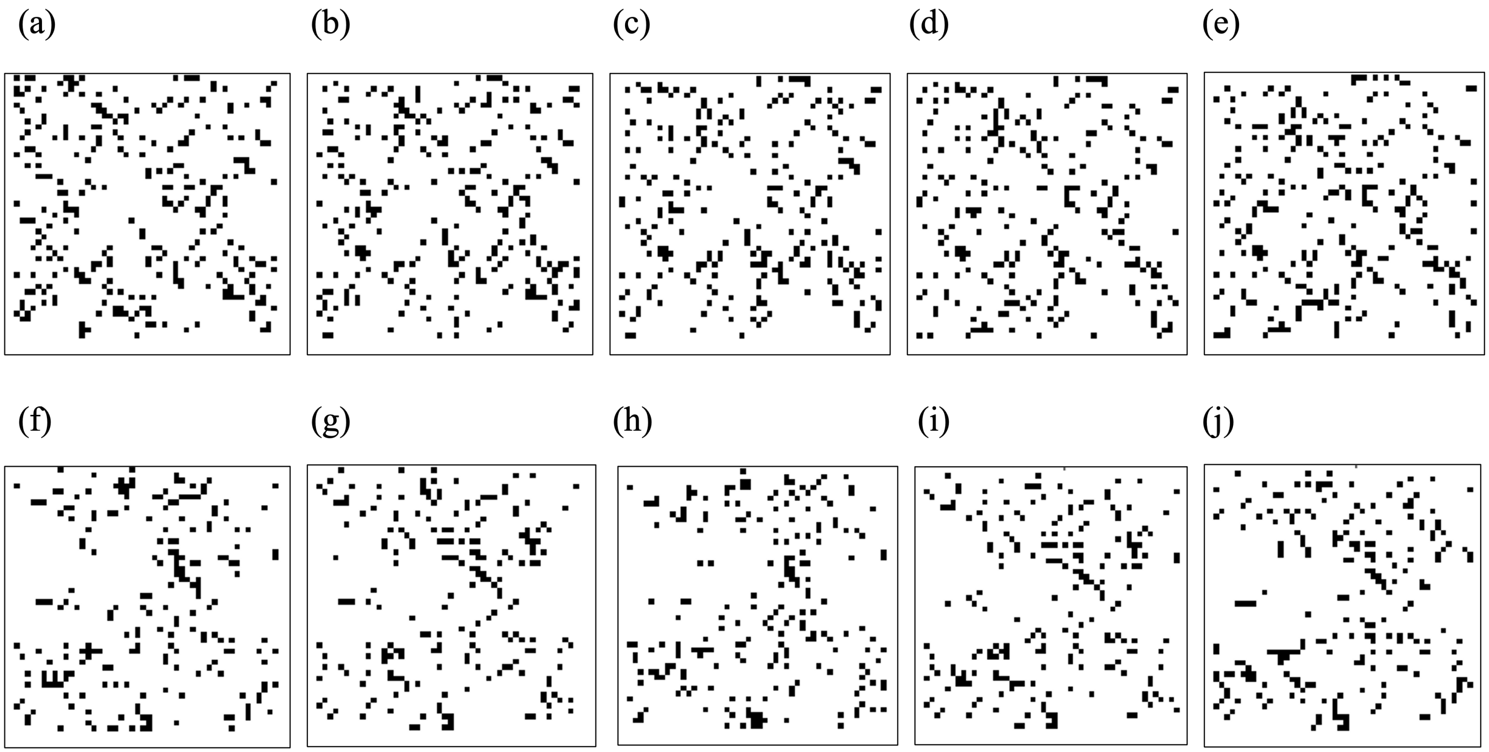

Figure S2: Stress maps at the intrinsic interface at (a-e) T = 0.8 and, (f-j) T = 1.0 at different time steps.

Percolation network:

The concept of percolation being a probabilistic feature, no stress range guarantees that a system shall percolate all the time. But closer to the percolating threshold, majority of the configurations (at least 50 percent) percolate, whereas, away from the threshold, although spanning cluster may form, such event is infrequent. In Fig. S3, the configurations shown in the top panels (a-h) are for an interface at T = 0.8 for stresses $[\infty, -3.5]$, where percolating network is observed majority of the time. However, at some instances, i.e., in (b) and (d) the clusters marginally fail to percolate in both directions. In contrast, for the same stress range, and as shown in the bottom panels (g-l), an interface at $T = 1.0$ percolates very rarely, as in panel (k), and in most cases, smaller clusters are formed reflecting the greater stress heterogeneity of the surface.

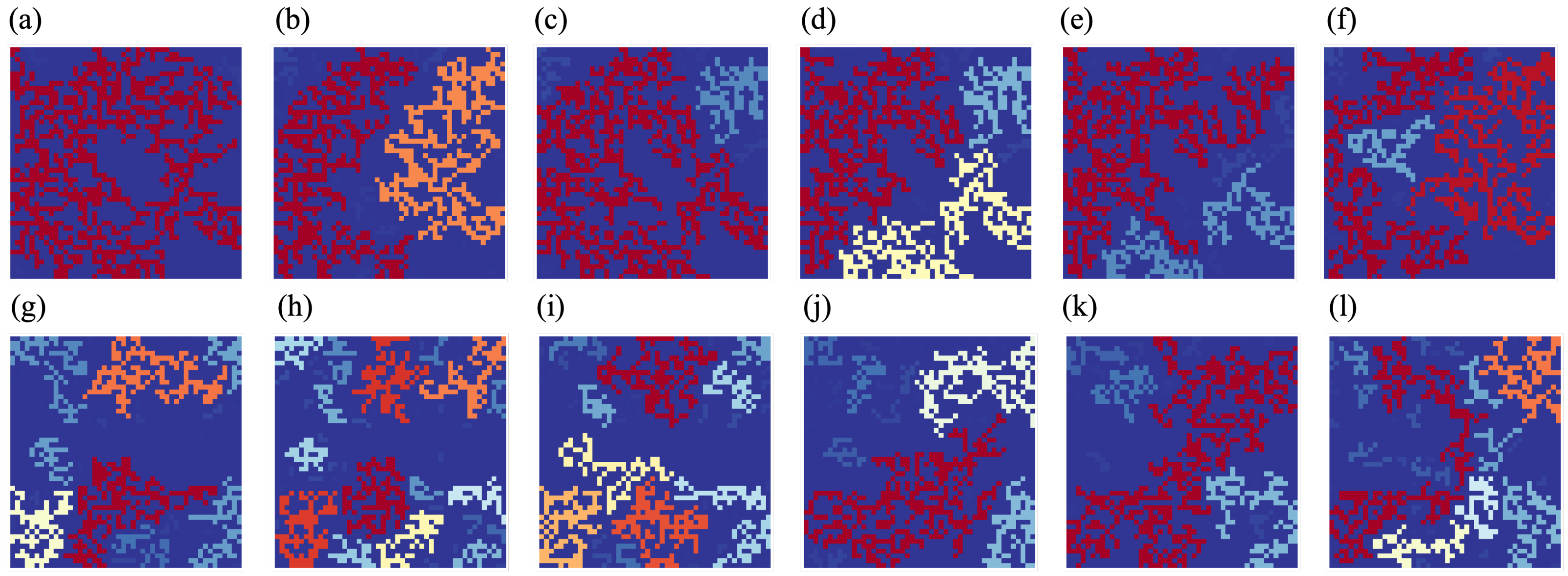

Figure S3: Stress clusters at the intrinsic interface at (a-g) T = 0.8 and, (h-l) T = 1.0 for stresses $[\infty, -3.5]$. In the top panel, all shown configurations except those in (b) and (d) percolate, whereas, in the bottom panel, none but the configuration in (k) percolates.

Liquid-vapor coexistence

The densities of the bulk vapor and liquid phases, and the pressure obtained from current simulation are tabulated in table S1 with the corresponding data from NIST.[1]

Table S1: Liquid-vapor coexistence data

T	$\rho_{\mathrm{l,present}}$	$\rho_{\mathrm{l,NIST}}$	$\rho_{\mathrm{v,present}}$	$\rho_{\mathrm{v,NIST}}$	P^N_{present}	$P^{\mathrm{sat}}_{\mathrm{NIST}}$
0.7	0.833	0.820	0.00302	0.0035	0.0020	0.0023
0.75	0.810	0.797	0.0058	0.0064	0.0039	0.0043
0.8	0.786	0.772	0.0110	0.0100	0.0068	0.0074
0.9	0.739	0.720	0.0229	0.0230	0.0155	0.0179
1.0	0.681	0.659	0.0402	0.0466	0.0289	0.0363

References

(1) *SAT-TMMC: Liquid-Vapor Coexistence Properties - Linear-Force Shifted Potential at* $4.0\,\sigma$; National Institute of Standards and Technology, accessed: 2022-04-16.
